# Circuit formation and sensory perception in the mouse olfactory system

**DOI:** 10.3389/fncir.2024.1342576

**Published:** 2024-02-16

**Authors:** Kensaku Mori, Hitoshi Sakano

**Affiliations:** ^1^RIKEN Center for Brain Science, Saitama, Japan; ^2^Department of Brain Function, School of Medical Sciences, University of Fukui, Matsuoka, Japan

**Keywords:** olfactory perception, neural-circuit formation, olfactory glomeruli, critical period, respiratory cycle, orthonasal / retronasal odors

## Abstract

In the mouse olfactory system, odor information is converted to a topographic map of activated glomeruli in the olfactory bulb (OB). Although the arrangement of glomeruli is genetically determined, the glomerular structure is plastic and can be modified by environmental stimuli. If the pups are exposed to a particular odorant, responding glomeruli become larger recruiting the dendrites of connecting projection neurons and interneurons. This imprinting not only increases the sensitivity to the exposed odor, but also imposes the positive quality on imprinted memory. External odor information represented as an odor map in the OB is transmitted to the olfactory cortex (OC) and amygdala for decision making to elicit emotional and behavioral outputs using two distinct neural pathways, innate and learned. Innate olfactory circuits start to work right after birth, whereas learned circuits become functional later on. In this paper, the recent progress will be summarized in the study of olfactory circuit formation and odor perception in mice. We will also propose new hypotheses on the timing and gating of olfactory circuit activity in relation to the respiration cycle.

## Introduction

Sensory systems are generated by a combination of activity-dependent and -independent processes. The basic architecture of neural circuits is built before birth based on a genetic program without involving neuronal activity. However, spontaneous firing plays an important role in making the system functional (Yu et al., 2004; Lorenzon et al., 2015). In the muse olfactory system, intrinsic neuronal activity (Reisert, 2010) is needed to segregate glomerular structures for sensory map formation in the OB (Nakashima et al., 2019). After birth, odor-evoked activity further modifies the glomerular map to adapt to the environmental situation (Inoue et al., 2021). In the visual system, activity waves in the retina are required for the system to work (Espinosa and Stryker, 2012; Kirkby et al., 2013). Blocking of stimulation in a subset of neurons during the critical period results in permanent changes in the representation of the neurons (Hensch, 2005).

In the mouse olfactory system, there are two separate neural pathways, innate and learned, that transmit odor signals from the OB to the OC for odor perception and decision making (Kobayakawa et al., 2007). For instinctive decisions, olfactory information is directly conveyed by mitral cells (MCs) to distinct valence regions in the amygdala (Miyamichi et al., 2011; Root et al., 2014; Inokuchi et al., 2017). It has been hypothesized that for learned decisions, odor-map information is transmitted to the anterior olfactory nucleus (AON) by tufted cells (TCs) and then to the piriform cortex for odor perception, as well as for recollection of associated memory (Wilson and Sullivan, 2011; Russo et al., 2020; Mori and Sakano, 2021; Chen et al., 2022; Poo et al., 2022). It is assumed that recalled scene-memory further activates specific valence regions in the amygdala, which were connected to the memory engram (Josselyn and Tonegawa, 2020) in the previous experience (Motanis et al., 2014; Janak and Tye, 2015; Jin et al., 2015; East et al., 2021).

During development, innate circuits start to work around birth before the learned circuits become functional (Hall and Swithers-Mulvey, 1992). Although instinctive decisions are stereotyped, they can be modified or even changed by odor experience (Sullivan et al., 2000; Logan et al., 2012). During the critical period in neonates, the sensitivity to the imprinted odor is increased and positive quality is imposed on its memory (Inoue et al., 2021). Environmental odor inputs promote the recruitment of projection-neuron dendrites and synapse formation within the glomeruli (Liu, A. et al., 2016; Inoue et al., 2018). The primary projection of pioneer-OSNs establishes olfactory-map topography, relative glomerular locations, and glomerular sizes (Zou et al., 2009). However, OSNs are constantly replaced with newly-generated follower OSNs (Ma et al., 2014). Interneurons are also regenerated and synapse with the existing glomeruli (Bovetti et al., 2009). Thus, the olfactory perception is influenced by environmental odors even after the neonatal period (Geramita and Urban, 2016; Liu and Urban, 2017).

It is notable that olfactory perception and decision making appear to be related with respiratory phases (Mori and Sakano, 2021). In response to the environmental odors, external TCs (eTCs) are activated earlier in the inhalation phase, whereas MCs are activated later in the exhalation phase (Igarashi et al., 2012; Manabe and Mori, 2013; Ackels et al., 2020). Therefore, it is possible that learned decisions and instinctive decisions may be made independent of one another during the respiratory cycle. The olfactory system also appears to process the orthonasal and retronasal odors at different phases of respiration (Shepherd, 2012; Mori and Sakano, 2022a,b). Thus, the sampling of exteroceptive and interoceptive olfactory information may be discrete and not continuous. In mammals, there are two OB structures, right and left, each containing a pair of mirror-symmetric olfactory maps, lateral and medial. We previously proposed that each lateral and medial map may process orthonasal and retronasal odor information, respectively, and transmit odor signals to separate areas in the brain possibly using distinct neural pathways (Mori and Sakano, 2022a,b).

In this mini-review, we will discuss the recent progress in the study of olfactory circuit formation and odor perception, and propose new hypotheses for the timing and gating of sensory circuit activity.

### Olfactory map formation and odor recognition

One of the long-standing questions of olfaction was how the large diversity of odor information can be recognized with a limited number of *OR* genes (Mori and Sakano, 2011). There are ~100,000 volatile odorants and ~ 1,000 different odorant receptor (OR) species in mice (Buck and Axel, 1991). As each odor is composed of multiple odorants with different combinations and ratios, the number of odors is countless. Then, how is the vast diversity recognized in the mammalian olfactory system with a limited number of *OR* genes? The answer to this question is that odor signals are converted to a combinatorial pattern of activated glomeruli in the OB (Mori et al., 2006).

The glomerular map is generated as a result of axonal projection of OSNs to the OB. For this primary projection, there are two basic principles, “one neuron – one receptor rule” and “one glomerulus – one receptor rule” (Sakano, 2010). Each individual OSN expresses only one functional *OR*-gene allele by negative feedback regulation (Chess et al., 1994; Serizawa et al., 2000, 2003), and OSN axons expressing the same OR species converge to a particular glomerular structure (Mombaerts et al., 1996). As each glomerulus corresponds to a specific OR species, there are ~1,000 glomerular species in each of four olfactory maps in mice. Unlike antigen recognition in the immune system, ligand – receptor interactions are not strict: each receptor can recognize multiple odorant species, or vice versa. Moreover, as odor information is usually composed of multiple odorants with different ratios, odor signals detected in the olfactory epithelium (OE) are converted to a combinatorial pattern of firing glomeruli in the OB (Malnic et al., 1999). This odor-map pattern appears to allow the piriform cortex to identify and recognize the vast diversity of odor information (Wilson and Sullivan, 2011).

OSN projection is regulated by two major schemes: one is along the dorsal/ventral (D/V) axis, and the other is along the anterior/posterior (A/P) axis using separate sets of axon guidance molecules, e.g., Semaphorins (Sema), Plexins (Plxn), and Neuropilins (Nrp) (Sakano, 2020). D/V projection is mainly regulated by positional information of OSN cell-bodies in the OE (Astic et al., 1987; Cloutier et al., 2002; Takeuchi et al., 2010), whereas A/P projection is instructed by expressed OR species using cAMP as a second messenger (Imai et al., 2006). Non-neuronal intrinsic activity of ORs, whose levels are uniquely determined by OR species, is converted to cAMP and regulates the transcription levels of axon guidance molecules (Nakashima et al., 2013). The olfactory map is initially continuous and needs to be converted to a discrete map of glomeruli (Sengoku et al., 2001; Luo and Flanagan, 2007). Glomerular segregation takes place using two sets of axon-sorting molecules, adhesive (e. g., Kirrel2, Kirrel3, and BIG-2) and repulsive (e. g., Eph A and ephrin A), whose expression levels are determined by the OR specificity of OSNs (Serizawa et al., 2006; Kaneko-Gotoh et al., 2008). Although both A/P projection and glomerular segregation are instructed by the same OR specificity using cAMP as a second messenger, they are differentially regulated at separate stages of OSN development, immature and mature, using different types of G proteins, Gs and Golf, respectively (Nakashima et al., 2013).

### Synapse formation within the glomeruli

As mentioned in the previous section, topography of an olfactory map is established as a result of primary projection of OSNs. This process does not require the neuronal activity of OSNs. However, the map needs to become a discrete map segregating glomerular structures (Luo and Flanagan, 2007). Within each glomerulus, the basic synaptic structure is formed between the OSN axons and projection-neuron dendrites using intrinsic neuronal activity (Yu et al., 2004; Lorenzon et al., 2015; Nakashima et al., 2019). Thus, the basic structure of a naïve glomerular map is stereotyped, although there are some differences among individuals due to the genetic polymorphism in the OR-multigene family (Keller et al., 2007).

**Figure 1:**
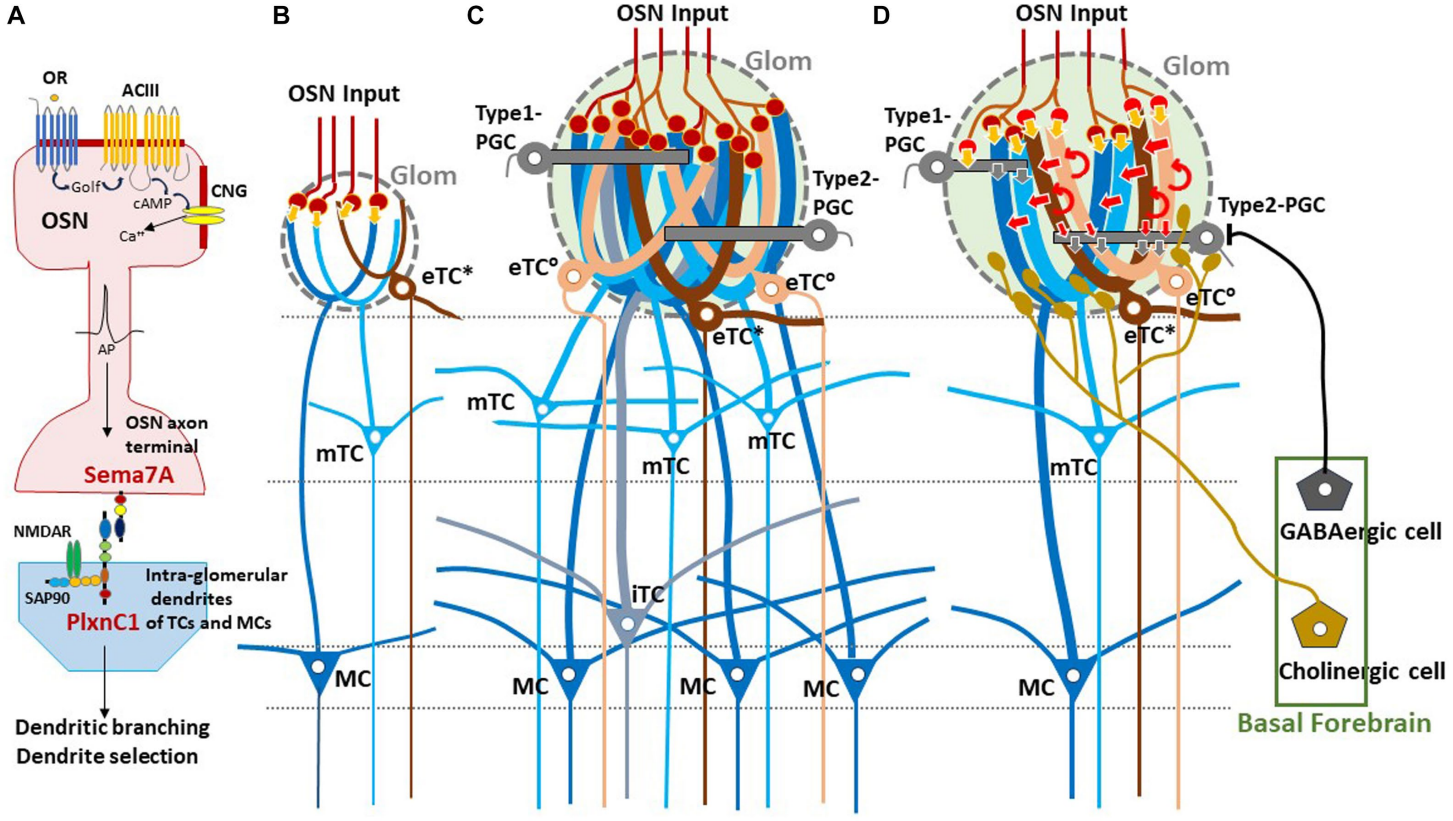
Experience-dependent development of glomerular circuitry in neonates. **(A)** Sema7A-PlxnC1 interaction essential to the induction of activity-dependent formation of glomerular circuitry during the critical period. ACIII, class III adenylyl cyclase; AP, action potential; cAMP, cyclic adenosine monophosphate; CNG, cyclic-nucleotide-gated channel; Golf, olfactory G protein; NMDAR, N-methyl-D-aspartate receptor; OR, odorant receptor; OSN, olfactory sensory neuron; PlxnC1, Plexin C1; SAP90, synapse-associated protein 90; Sema7A, Semaphorin 7A. **(B,C)** Schematic diagrams of an undeveloped glomerular structure **(B)** and a fully developed glomerulus **(C)**. Note that the fully developed glomerulus recruits apical dendrites of numerous projection neurons and inhibitory interneurons. **(D)** Synaptic organization within the fully developed glomerulus. In **(B–D)**, five types of projection neurons are illustrated; external tufted cells without lateral dendrites (eTC°, peach), external tufted cells with lateral dendrites (eTC*, brown), middle tufted cells (mTC, light blue), internal tufted cells (iTC, gray blue), and mitral cells (MC, dark blue). These cells receive direct inputs from OSN axons (orange arrows) and indirect input via eTCs (red arrows) in the glomerulus (Glom). Curved red arrows show self-excitation of eTCs via dendro-dendritic excitatory synapses. Periglomerular cells (PGCs) are classified into Type 1 and Type 2. OSN axons form direct excitatory synapses on Type 1 PGCs but do not form synapses on Type 2 PGCs. GABAergic cells of the basal forebrain form inhibitory synapses on Type 2 PGCs but do not form synapses on Type 1 PGCs. Cholinergic cells in the basal forebrain project to all types of projection neurons and to Type 2.3 PGCs (a subset of Type 2 PGCs).

In neonates, the olfactory map is further modified by odor-evoked OSN activity during the critical period. This plastic change of glomeruli known as olfactory imprinting is triggered by a signaling molecule, Sema7A, expressed in OSN axons (Inoue et al., 2018, 2021). Interactions of Sema7A with its receptor PlxnC1 localized to the projection-neuron dendrites initiate a series of postsynaptic events that promote dendrite selection and maturation (Inoue et al., 2018) (Figure 1A). Odor-evoked enhancement of synapse formation enlarges the glomerular sizes, increasing the number of recruited dendrites of projection neurons and periglomerular cells (PGCs) (Liu et al., 2016; Inoue et al., 2018) (Figures 1B–D). It is notable that the number of projecting OSN axons does not change by imprinting.

Sema7A expression in OSNs is activity-dependent and totally abolished in the knock-out (KO) of cyclic-nucleotide-gated (CNG) channels (Inoue et al., 2018). Thus, the activity-dependency of olfactory imprinting is supported by Sema7A. In contrast, PlxnC1 expression does not require the neuronal activity, but dendrite localization of PlxnC1 is restricted to the first week after birth. Therefore, PlxnC1 determines the time frame of the olfactory critical period. Olfactory imprinting not only increases the sensitivity to the imprinted odor, but also imposes the positive quality on imprinted memory. In the KO studies and rescue experiments, oxytocin was found to be responsible for this quality change of olfactory memory (Inoue et al., 2021). In the oxytocin KO, the sensitivity to the imprinted odor is increased but the attractive quality is not imposed, showing the defects in the social memory test as adults. When oxytocin is administrated by intraperitoneal injection, this impairment is rescued if the mice are treated with oxytocin in neonates (Inoue et al., 2021). It has been reported that olfactory perception can be changed by environmental odors even after the neonatal critical period affecting social responses as adults (Gur et al., 2014; Muscatelli et al., 2018). However, it is yet to be studied, what kind of signaling system is involved in this postnatal adaptation.

### Projection neurons and decision making

The olfactory map is not merely a projection screen to display a pattern of activated glomeruli, but is also composed of distinct functional domains for innate odor qualities (Kobayakawa et al., 2007; Liberles, 2015). A pattern of an odor map appears to be recognized as a whole to identify the input odor and to recollect the odor memory of associated scenes (Mori and Sakano, 2021). It has been reported that odor information is transmitted from the OB to the AON by eTCs, preserving the odor-map topography (Schoenfeld and Macrides, 1984; Yan et al., 2008; Grobman et al., 2018; Hirata et al., 2019), and then to the piriform cortex for olfactory perception (Russo et al., 2020).

We hypothesized that using an odor map as a two-dimensional QR code, the associated memory engram may be searched for, and then the odor scene may reactivate the valence circuit that was connected to the memory engram in the previous odor experience (Mori and Sakano, 2021).

In contrast to the neural pathway for learned decisions, the innate pathway is simpler in the mouse olfactory system: Odor information is directly transmitted from a particular functional domain in the OB to the valence region in the amygdala using another type of projection neuron, MCs (Miyamichi et al., 2011; Root et al., 2014; Inokuchi et al., 2017). It has been shown that innate behavioral responses can be induced even by a single glomerular species. For example, if the Olfr1019 glomerulus for the fox odor TMT (trimethyl thiazoline) is photo-activated, an immobility response (freezing) is elicited, although stress-induced aversive responses (Kondoh et al., 2016) are not induced and plasma concentrations of the stress hormone ACTH does not increase (Saito et al., 2017).

Olfactory quality is roughly sorted into two separate types, aversive and attractive during the process of OSN projection (Horio et al., 2019). Aversive odor information collected in the dorsal subdomain D_I_ (Kobayakawa et al., 2007) is further transmitted to the postero-medial (pm) cortical amygdala (CoA) (Miyamichi et al., 2011; Root et al., 2014). In contrast, attractive social information in the ventral OB (Lin et al., 2005) is delivered to the anterior medial-amygdala (aMeA) (Inokuchi et al., 2017). However, the spatial segregation for encoding the innate odor valence is still controversial, because the CoA is reported to carry both appetitive and aversive valence for odors (Root et al., 2014; Lurille and Datta, 2017). The dorsal subdomain D_III_ for TAAR (trace amine-associated receptor) contains mostly aversive glomeruli. However, it also contains some appetitive ones (Liberles, 2015). Activation of dorsal glomeruli may not necessarily trigger aversive responses and activation of ventral glomeruli may not always trigger attractive responses (Qiu et al., 2021).

There are two distinct MC pathways, aversive and attractive, that are segregated by the Nrp2/Sema3F guidance system. MCs, both Nrp2^+^ and Nrp2^−^, originate in the ventricular zone in the embryonic OB and migrate radially to the MC layer (Imamura et al., 2011; Inokuchi et al., 2017). The Nrp2^+^ MCs further migrate tangentially to the ventral region as the OB structure expands. The repulsive ligand Sema3F secreted by the early-arriving Nrp2^−^ OSN axons in the dorsal OB pushes down the Nrp2^+^ MCs to the ventral region in the MC layer of the OB. This Sema3F also guides the late-arriving Nrp2^+^ OSN axons to the ventral glomerular layer in the OB.

For synapse formation, primary dendrites of projection neurons are connected to the nearest neighboring glomeruli regardless of their OR specificity (Nishizumi et al., 2019). Therefore, it is important for OSNs to project their axons to a correct site in the glomerular layer in the OB, and for projection neurons to migrate to an appropriate position in the MC layer. Common usage of the same signaling system of Nrp2/Sema3F in both OSN projection and MC migration appears to be important for proper matching of OSN axons and MC dendrites for synapse formation. Axon guidance of Nrp2^+^ MCs to the aMeA is also mediated by Sema3F, but by separate Sema3F expressed in the OC (Inokuchi et al., 2017).

In the Nrp2 KO for MCs, social responses, e.g., male–female attraction and pup suckling, are perturbed (Inokuchi et al., 2017). Rescue experiments indicate that a single signaling system, Nrp2/Sema3F, is sufficient for the segregation of the attractive and aversive olfactory circuits (Inokuchi et al., 2017). *In utero* electroporation of the human *Nrp2* (*hNrp2*) into the embryonic OB demonstrates that ectopic expression of the *hNrp2* gene alone can change the fate of Nrp2^−^ MCs: OCAM-positive dorsal-MCs that are supposed to project to the pmCoA are brought to the ventral OB and further guided to the aMeA by hNrp2.

### Direct and indirect pathways within the glomerulus

Glomeruli are the site of odor information transmission from OSNs to several types of projection neurons in the OB (Figure 1). The projection neurons are functionally and morphologically classified into external tufted cells (eTCs), middle tufted cells (mTCs), internal tufted cells (iTCs), and mitral cells (MCs) (Shepherd et al., 2004; Nagayama et al., 2014; Mori and Sakano, 2022a). Each type of projection neuron has a distinct pattern of lateral dendrite projection in the external plexiform layer (EPL) and axonal projection to the OC (Igarashi et al., 2012). External tufted cells are further classified into two subpopulations; one subpopulation (eTC^°^) lacks lateral dendrites and the other subpopulation (eTC*) has lateral dendrites in the most superficial part of the EPL (Hayer et al., 2004; Antal et al., 2006).

In the glomeruli of adult rodents, the external odor information carried by OSNs is transferred to mTCs and MCs via two types of pathways: direct mono-synaptic pathway from OSN axons, and indirect multi-synaptic pathway first from OSN axons to eTCs and then from eTCs to mTCs and MCs (Figure 1) (Najac et al., 2011; Gire et al., 2012). In the first step of the indirect pathway, the synaptic input from OSNs induces synchronized burst firings of several eTCs belonging to the same glomerulus during the inhalation phase. The synchronized burst discharges of these eTCs then activate mTCs and MCs of the same glomerulus via dendrodendritic excitatory synapses within the glomerulus.

One important question of glomerular circuit development is whether early-life olfactory experiences play a role in the formation of the direct and indirect pathways. Early olfactory experiences enlarge the sizes of activated glomeruli, although the number of projecting OSNs stays the same (Inoue et al., 2018, 2021). Because the adult OB contains glomeruli with a variety of sizes (Mori, 2014), we propose a hypothesis that the size of a glomerulus expands in proportion to the amount of early-life olfactory inputs to the glomerulus during the neonatal critical period, i.e., the first week after birth. Enlarged glomeruli may have experienced more early-life olfactory inputs and recruited numerous apical dendritic branches of eTCs, mTCs, and MCs.

We hypothesize that these large glomeruli recruit many eTCs and thus form the robust indirect OSNs→eTCs→mTCs/MCs pathway in addition to the direct pathway (Figures 1C,D). It should be noted that pairing an odor with aversive stimulus can also lead to enlargement of glomeruli in adults (Jones et al., 2008) and that olfactory extinction training reverses the increase in glomerular size (Morrison et al., 2015).

We also speculate that micro- or small-glomeruli (Lipscomb et al., 2002) are ones that experienced no or little early-life olfactory inputs and failed to recruit an adequate number of eTC, mTC, and MC dendritic branches, resulting in the lack of the functional indirect pathway (Figure 1B). These undeveloped glomeruli may contain only the direct pathway so that strong OSN inputs are required for inducing burst-firings of mTCs and MCs. The direct excitatory synapses from OSNs to these projection neurons can emerge before birth prior to the postnatal olfactory experiences.

OB slice experiments show that in the enlarged glomeruli, eTCs act as essential drivers of glomerular output, mediating feedforward transmission of the OSN input to mTCs and MCs (De Saint Jan et al., 2009, 2022). It has been hypothesized that MCs are not typically activated by the direct OSN input and instead require the multi-synaptic indirect pathway via eTCs (Gire et al., 2012). The eTCs have the lowest odor concentration threshold for inducing firing response (Igarashi et al., 2012), generate self-regenerative long-lasting depolarization that function as a bimodal on/off switch (Gire and Schoppa, 2009), and interact with each other through their dendrodendritic excitatory synapses. Therefore, once some eTCs are activated by the OSN input, many eTCs of a same glomerulus may be entrained to generate synchronized burst firings for a fixed duration, suggesting that the indirect pathways act as a low threshold booster of the OSN signal.

### Respiration phase-coherent activity of OB neurons

Recordings of local field potentials and spike activities of OB neurons in awake rats and mice indicate that, in response to odor inhalation, projection neurons in the lateral map show burst firings during specific time windows in the respiration cycle and transmit external odor information sequentially to the OC (Briffaud et al., 2012; Igarashi et al., 2012; Manabe and Mori, 2013; Mori et al., 2013; Nagayama et al., 2014; Short et al., 2016; Díaz-Quesada et al., 2018; Ackels et al., 2020; Eiting and Wachowiak, 2020; Mori and Sakano, 2021). What is the neural circuit mechanism that allows different types of projection neurons to respond during different time windows of the respiration cycle?

How does the indirect pathway via eTCs contribute to the generation of sequential activation?

**Figure 2:**
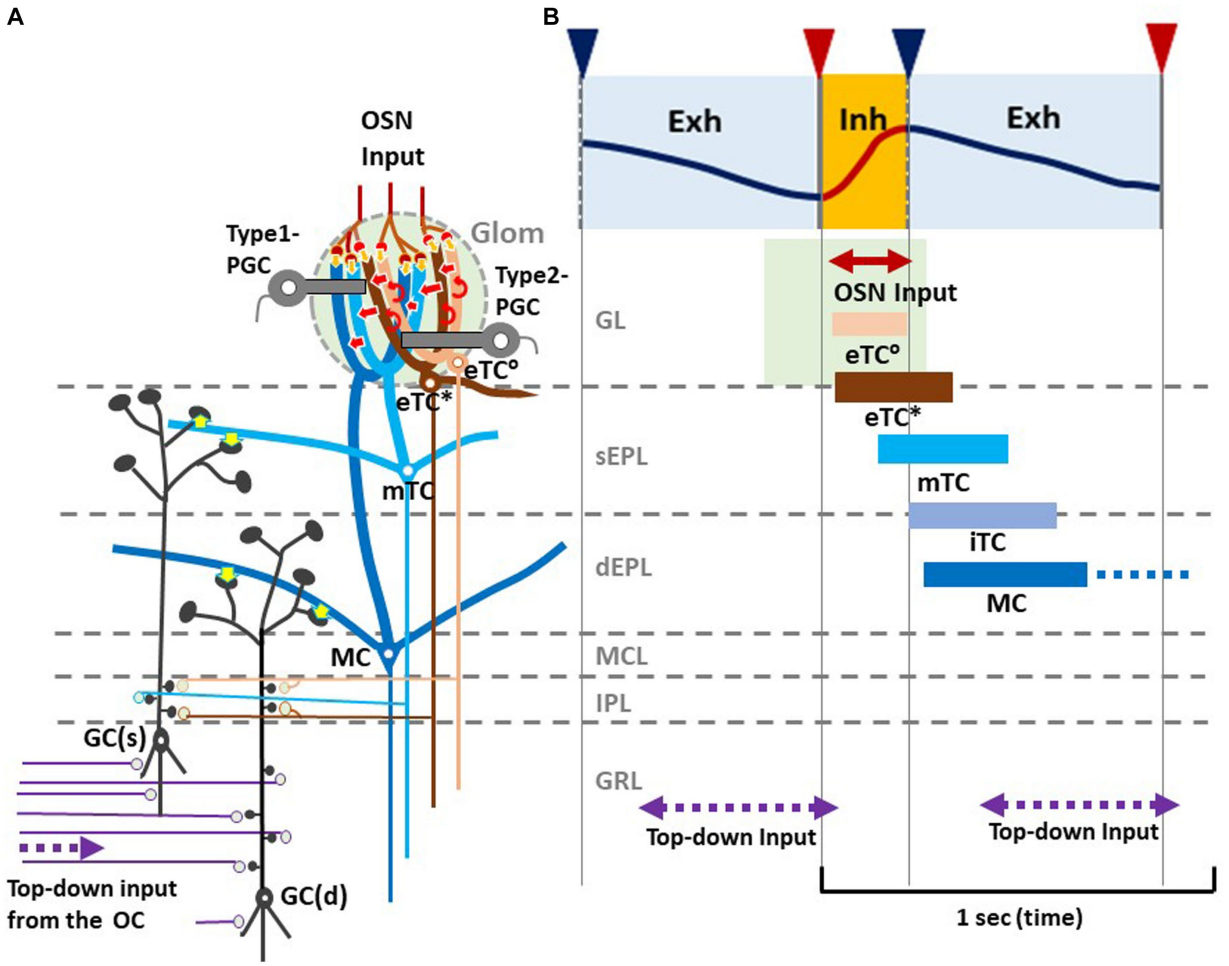
Respiration-phase-coherent neural activity in the olfactory bulb **(A)** Schematic of basic synaptic organization within the glomerulus (Glom), external plexiform layer (EPL), internal plexiform layer (IPL), and granule cell layer (GRL) of the OB. For simplicity, only four types of projection neurons (eTC°, eTC*, mTC, and MC) and two types of inhibitory interneurons, PGCs and granule cells (GCs), are shown. eTC*s and mTCs project lateral dendrites to the superficial part of the external plexiform layer (sEPL) and form dendro-dendritic excitatory synapses (yellow arrows in sEPL) on spines of GC(s) that project dendrites to the sEPL. MCs project lateral dendrites to the deep part the external plexiform layer (dEPL) and form dendro-dendritic excitatory synapses (yellow arrows in dEPL) on spines of GC(d) that send dendrites to the deep part. **(B)** Respiration-phase coherent neural activity during quiet wakefulness in the rat OB. Upper most trace shows the respiration monitored by a thermistor placed in the nasal cavity. Upward swing indicates inhalation (magenta line and orange shaded) and downward swing exhalation (blue line and blue shaded). Magenta triangles indicate the onset of inhalation, and blue triangles show the onset of exhalation. In response to the inhalation of room air, projection neurons respond sequentially with burst firings in the respiration cycle. Magenta double-arrow line indicates the time window of OSN input. Peach bar shows the time window of burst firings of eTC°s. Brown bar indicates the time window of burst firing of eTC*s that project lateral dendrites to the most superficial part of the external plexiform layer (EPL). Light blue bar shows the time window of firings of mTCs that send lateral dendrites to the superficial sublayer of the EPL (sEPL). Gray blue bar indicates the time window of firing of iTCs that send lateral dendrites to the middle part of the EPL. Dark blue bar shows the time window of firings of MCs that send lateral dendrites to the deep sublayer of the EPL (dEPL). Purple double-arrow dashed lines indicate the time window of top-down input from the olfactory cortex (OC) to the granule cell layer (GRL) of the OB. Green-shaded area indicates the time window of possible depolarization of primary dendrites of projection neurons in the glomerular layer (GL).

As illustrated in Figure 2B, during the awake resting state, eTCs of activated glomeruli show synchronized burst firings during the period from the onset of odor inhalation to the initial part of exhalation (eTC^°^: peach bar, and eTC*: brown bar), transmitting odor information to the pars externa of the anterior olfactory nucleus (AONe) and the most anterolateral isolation of the CAP compartments (aiCAP) of the olfactory tubercle (Hirata et al., 2019). eTC circuits generate earliest-onset highest-frequency burst firings during the odor inhalation phase. mTCs of the activated glomeruli show synchronized high-frequency burst firings during the period from the middle of inhalation to the early 1/3 of exhalation (light blue bar), transmitting the odor information to olfactory peduncle areas. Because eTCs and mTCs of the lateral map send axons to specific areas of the AON, they appear to be the origin of the multi-synaptic learned decision pathway, transmitting external odor information to higher cognitive centers via AON and APC, mainly during the inhalation phase (Mori and Sakano, 2021). iTCs of activated glomeruli generate synchronized burst firings during the period from the end of inhalation to the early 2/3 of exhalation (gray blue bar), transmitting odor information to wide areas of the OC. MCs show synchronized burst firings during the period from the start of exhalation to the early 2/3 of exhalation (dark blue bar), transmitting odor information to widespread areas of the OC. A subset of these MCs continues burst firings up to the onset of the next inhalation (dashed dark blue bar). Thus, iTC and MC circuits show later-onset low-frequency burst firings that last into the early 2/3 of the exhalation phase. A subset of MCs forms a direct mono- synaptic pathway, sending innate behavioral decision signals to the cortical and medial amygdaloid nuclei (Mori and Sakano, 2021).

Thus, we propose a hypothetical model that in response to odor inhalation, eTCs, mTCs, iTCs and MCs respond sequentially with synchronized burst firings, each transmitting distinct information at a specific time window within the respiratory cycle. Each type of projection neuron sends lateral dendrites to a specific sublayer of the EPL and form dendrodendritic excitatory synapses on granule cells (inhibitory interneurons) (Figure 2A, yellow arrows). eTC*s and mTCs project their lateral dendrites to the superficial sublayer of the EPL and form dendrodendritic excitatory synapses on a subset of granule cells [GC(s)] that send apical dendrites to the superficial sublayer.

MCs project lateral dendrites to the deep sublayer of the EPL and form dendrodendritic excitatory synapses on a different subset of granule cells [GC(d)] that project apical dendrites to the deep sublayer (Mori, 1987). These connectivity patterns suggest that each subset of granule cells receive dendrodendritic synaptic inputs from selective types of projection neurons at a specific time window in the respiratory cycle.

In addition to the respiration phase-coherent burst firings of projection neurons, top-down inputs from the OC to granule cells appear to occur at a specific time window in the respiration cycle (Figures 2A,B). Analysis of local field potentials in the OB of awake rats indicates that top-down information flow from the OC to the granule cells occurs predominantly during the time window from the early 1/3 to the end of the exhalation phase. Thus, respiration-phases coordinate the timing of odor information processing by different local circuits in the OB, the timing of odor signal transmission to the OC by different types of projection neurons, and the timing of top-down signal transmission from the OC to the OB.

### Modulation of glomerular activity by periglomerular cells and basal forebrain

Sensory experience-dependent formation of neural circuits requires balanced excitation-inhibition (Yazaki-Sugiyama et al., 2009). PGCs are local inhibitory interneurons that project dendritic branches to the glomerulus and interact with the excitatory projection neurons within the glomerulus. Early olfactory experiences also recruit PGC dendrites to activated glomeruli (Inoue et al., 2021). PGCs are classified into two subtypes (Kosaka and Kosaka, 2005). Type 1 PGCs receive direct excitatory synaptic inputs from OSNs but not from GABAergic inhibitory cells of the basal forebrain (BF, magnocellular preoptic area), whereas Type 2 PGCs do not make OSN synapses but receive axonal input from the BF GABAergic cells (Figure 1D) (Sanz Diez et al., 2019; De Saint Jan, 2022).

In addition to the BF GABAergic cells, cholinergic cells in the horizontal limb of the diagonal band of the BF densely innervate the glomerular circuits (Zaborszky et al., 1986; Bendahmane et al., 2016). While the BF GABAergic inhibitory axons target only Type 2 PGCs, the BF cholinergic axons target apical dendrites of all types of projection neurons including eTCs and Type 2.3 PGCs (a subset of Type 2 PGCs) (Spindle et al., 2018; De Saint Jan, 2022) (Figure 1D). BF cholinergic cells are tonically active during wakefulness but silent during slow-wave sleep (Xu et al., 2015). We assume that, during wakefulness, arousal signals of BF cholinergic cells may induce tonic depolarization in the dendritic tufts of projection neurons especially those of eTCs (D’Souza et al., 2013), facilitating the transmission of OSN inputs via the indirect pathway to mTCs and MCs. In contrast, during slow-wave sleep the lack of cholinergic input to eTCs may hyperpolarize eTCs and thus prevent OSN inputs to be transmitted via the indirect pathway to the projection neurons. In agreement with this idea, in freely behaving rats, local field potentials that reflect burst firings of projection neurons occur consistently in response to each inhalation of room air during wakefulness, whereas these local field potentials diminish or disappear during slow-wave sleep (Manabe and Mori, 2013).

During wakefulness, BF cholinergic input activates Type 2.3 PGCs via muscarinic acetylcholine receptors, such that Type 2.3 PGCs fire tonically (De Saint Jan, 2022). The tonic firing of Type 2.3 PGCs causes tonic inhibition of eTCs in the indirect pathway. Because GABAergic cells in the medial septum and vertical limb of the diagonal band in the BF show burst firings that are coherent to specific time frames of the respiration cycle, thus sending respiration phase-specific attention signals to the hippocampal circuits during exploratory behavior (Tsanov et al., 2014), we propose a hypothesis that individual GABAergic cells in the BF generate burst firings and send inhibitory output to Type 2.3 PGCs during a cell-specific time frame in the respiration cycle.

We hypothesize that BF GABAergic cells that project to the lateral map glomeruli generate burst discharges selectively during intended inhalation phase, and thus inhibit Type 2.3 PGCs and disinhibit eTCs. By the respiration phase-specific gating of the indirect pathway, the lateral map glomeruli are ready to boost orthonasal/exteroceptive odor inputs during the inhalation phase, whereas they shut out the OSN input during the exhalation phase. We also hypothesize that BF GABAergic cells that project to the medial map glomeruli generate burst discharges selectively during intended exhalation phase, inhibiting Type 2.3 PGCs and disinhibiting eTCs. By the gating of the indirect pathway, the medial map glomeruli are ready to enhance retronasal/interoceptive odor inputs during the exhalation phase, whereas they shut out the OSN input during the inhalation phase.

## Discussion

During development, an olfactory map in the OB is formed based on a genetic program without involving neuronal activity. Agonist-independent receptor activity of GPCRs is involved in OR-instructed OSN projection using cAMP as a second messenger (Imai et al., 2006; Nakashima et al., 2013). An olfactory map is initially continuous, but becomes discrete, segregating glomerular structures in an activity- dependent manner (Serizawa et al., 2006; Luo and Flanagan, 2007). Intrinsic neuronal activity of OSNs appears to be responsible for this OR-specific glomerular segregation (Serizawa et al., 2006; Nakashima et al., 2019). The naïve glomerular map is further modified by odor-evoked OSN activity during the neonatal critical period. When the pups are exposed to a particular odorant, responding glomeruli increase their sizes by recruiting dendrites of projection neurons, as well as of interneuron PGCs. Sema7A/ PlxnC1 signaling is responsible for this promotion of post-synaptic events within the glomeruli (Inoue et al., 2018). Glomerular enlargement increases the sensitivity to the imprinted odor and this imprinting imposes the attractive quality on odor memory, even when the odor quality is innately aversive (Inoue et al., 2021). Environmental odors continue to affect odor perception even after the neonatal critical period (Sullivan et al., 2000; Liu and Urban, 2017). However, signaling systems responsible for this postnatal odor-adaptation need to be clarified by future experiments.

We assume that the OB of an individual adult-mouse has a unique combination of enlarged glomeruli, which reflects the early-life olfactory experience. Each enlarged glomerulus may form the imprinted memory-circuit of odor-evoked OSN signals. It is known that there are two symmetrical olfactory maps, lateral and medial, in each right and left OBs. We speculate that the enlarged glomeruli in the lateral map may represent neonatal experiences of orthonasal/exteroceptive odors, while those glomeruli in the medial map may exemplify early-life retronasal/interoceptive inputs.

Odor information is processed by two distinct olfactory circuits, innate and learned (Kobayakawa et al., 2007). For learned decisions, we assume that combinatorial signals of activated glomeruli formed in the OB are transmitted to the piriform cortex via the AON and utilized for identification of odor information and recollection of an associated memory-scene (Russo et al., 2020; Mori and Sakano, 2021; Poo et al., 2022). For innate decisions, stimulated MC signals from a particular functional domain in the olfactory map are directly transmitted to specific valence regions in the amygdala (Miyamichi et al., 2011; Root et al., 2014; Inokuchi et al., 2017). As TCs are activated earlier in the inhalation phase and MCs are activated later in the exhalation phase, we speculate that learned and innate decisions may separately be made during the respiratory cycle (Mori and Sakano, 2022a).

We previously proposed another hypothesis that each lateral and medial map in the OB may separately process orthonasal/exteroceptive and retronasal/interoceptive odor information, respectively (Mori and Sakano, 2022b). Here we further propose that the BF GABAergic input to the glomerular circuit is respiration-phase coherent and gates the orthonasal and retronasal odor inputs. The BF GABAergic input to the lateral-map glomeruli appears to occur during the intended inhalation phase, resulting in the inhalation-phase specific boosting of the orthonasal inputs. The BF GABAergic input to the medial map glomeruli may occur during the intended exhalation phase, resulting in the exhalation phase-specific amplification of the retronasal input. The BF GABAergic cells that target GCs (Villar et al., 2021) may also fire in a respiration-phase coherent manner.

Respiration-phase coherent neural-circuit activity occurs throughout the brain including the neocortex, hippocampus, olfactory cortex, thalamus, and cerebellum (Ito et al., 2014; Moberly et al., 2018; Tort et al., 2018; Bagur et al., 2021; Girin et al., 2021; Karalis and Sirota, 2022; Folschweiller and Sauer, 2023), as well as the respiratory central-pattern generator areas of the brainstem (Krohn et al., 2023). In the exploratory behavior of rodents, nose motion, head bobbing, and whisking are synchronized with respiration phases such that the inhalation phase corresponds to the time window of multisensory sampling of environmental information (Kurnikova et al., 2017). The subsequent exhalation phase may provide the time window for the brain to evaluate the sampled sensory information and express the behavioral and emotional outputs.

It has been suggested that the respiration phase coordinates with the timing of information transfer across wide-spread regions in the brain for multisensory cognition (Folschweiller and Sauer, 2023). The experience-dependent development of the glomerular circuitry and the behavioral state- and respiration phase-dependent modulation of circuit function in the OB will provide us with an excellent model system for understanding the mechanism of dynamic orchestration of neural circuits in the brain. We hope that our hypotheses proposed in this mini-review will be of help in clarifying the multisensory cognition and decision making in mammals.

## Author contributions

Both authors contributed equally in writing this article.

## Glossary

aiCAP: The most anterolateral isolation of the CAP compartments of the olfactory tubercle
aMeA: Anterior medial-amygdala
AON: Anterior olfactory nucleus
A/P: Anterior/Posterior
BF: Basal forebrain
D/V: Dorsal/Ventral
EPL: External plexiform layer
eTC: External tufted cell
GC: Granule cell
iTC: Internal tufted cell
MC: Mitral cell
mTC: Middle tufted cell
Nrp: Neuropilin
OB: Olfactory bulb
OC: Olfactory cortex
OE: Olfactory epithelium
OR: Odorant receptor
OSN: Olfactory sensory neuron
PGC: Periglomerular cell
Plxn: Plexin
pmCoA: Postero-medial cortical amygdala
Sema: Semaphorin
TC: Tufted cell
TMT: Trimethyl thiazoline
